# Managing Ulcerative Colitis and Crohn’s Disease: Should the Target Be Endoscopy, Histology, or Both?

**DOI:** 10.1093/jcag/gwad034

**Published:** 2023-09-23

**Authors:** Fernando Magro, Maria Manuela Estevinho, André Valois

**Affiliations:** Department of Biomedicine, Unit of Pharmacology and Therapeutics, Faculty of Medicine, University of Porto, Porto, Portugal; CINTESIS@RISE, Department of Biomedicine, Faculty of Medicine of the University of Porto, Porto, Portugal; Department of Gastroenterology, São João Hospital Center, Porto, Portugal; Clinical Pharmacology Unit, São João Hospital University Center, Porto, Portugal; Department of Biomedicine, Unit of Pharmacology and Therapeutics, Faculty of Medicine, University of Porto, Porto, Portugal; Department of Gastroenterology, Vila Nova de Gaia Espinho Hospital Center, Vila Nova de Gaia, Porto, Portugal; Clinical Pharmacology Unit, São João Hospital University Center, Porto, Portugal

**Keywords:** inflammatory bowel disease, endoscopic outcomes, histological outcomes, remission

## Abstract

In inflammatory bowel disease (IBD), mucosal healing is the primary long-term treatment goal, encompassing both endoscopic and histological outcomes. This paper aims to overview the ability of new treatment options to promote endoscopic and histological healing and to discuss the prognostic significance of endoscopic and histological outcomes. The analysis included randomized-controlled trials (published since 2020) focused on the impact of pharmacological interventions on endoscopic and histological remission in IBD. Even though the Mayo endoscopic subscore is routinely used, the application of validated scoring systems for ulcerative colitis is uncommon. In Crohn’s disease (CD), the application of endoscopic scores remains limited to clinical studies. The standardized evaluation of histological features has been performed in several recent ulcerative colitis trials, resorting mostly to the Geboes score and the Nancy histological index. Still, the use of histological scores for CD remains elusive. Current evidence underscores that histological remission conveys the best long-term prognosis, supporting the inclusion of histology as a treatment guide in ulcerative colitis. In CD, data are promising but originated from a few retrospective studies. Further efforts are warranted to: (1) use validated histological indexes for ulcerative colitis, aiming their adoption as treatment targets; (2) promote the validation and utilization of histological scores for CD, at least in clinical studies; (3) confirm the prognostic impact of histological remission in CD; (4) integrate artificial intelligence assets to support grading, particularly in the setting of histology; (5) prospectively define the monitoring frequency of IBD patients who achieved histological remission.

## Introduction

Inflammatory bowel diseases (IBD), comprising Crohn’s disease (CD) and ulcerative colitis (UC), are chronic conditions characterized by inflammation of the gastrointestinal tract.^1,2^ Treatment targets for IBD have shifted over time from symptom control to achieving objective evidence of mucosal healing (MH), which encompasses both endoscopic and histological healing.^3,4^ In the setting of UC, MH has been associated with favourable long-term outcomes, including prolonged periods of corticosteroid-free remission and reduced risk of complications such as hospitalization, surgery, and colorectal cancer.^2^

In 2013, the International Organization for the Study of IBD established the STRIDE program^5^—Selecting Therapeutic Targets in IBD—to assist clinicians in setting appropriate treatment goals for different stages of IBD; the original STRIDE statements were revised in 2021 to include treatment targets in both adult and pediatric IBD.^6^ The STRIDE program recommends a treat-to-target (T2T) approach based on evidence and expert consensus, including clinical indexes, biochemical biomarkers, endoscopic healing, and histological healing in the case of UC. All these goals are viewed as complementary and are integrated into the concept of disease clearance.^4^

In IBD, achieving MH is the primary long-term treatment goal. In this setting, endoscopic remission assessed through endoscopic indexes has proved to be a key target associated with improved long-term outcomes.^2^ However, a significant proportion of patients who achieve endoscopic healing may still present microscopic disease.^7,8^ In UC patients, histological activity, even in the presence of endoscopic remission, is associated with higher relapse rates, prolonged corticosteroid use, and long-term complications.^9^ Therefore, histological assessment using histological indexes has been proposed as a more accurate measure of disease activity. Recent international consensus has recognized that histological remission is an appropriate endpoint in both UC and CD clinical trials.^10,11^ However, histological healing has not yet been formally endorsed as a treatment goal in clinical practice, partly due to the existence of multiple unvalidated histological scoring systems.^6^ Furthermore, we are witnessing a growing interest in the use of molecular targets to evaluate IBD, including markers of inflammation, immune activation, and mucosal damage, which have shown utility in predicting disease activity and treatment response.^6^

The aim of this review was to discuss the evaluation of endoscopic and histological activity in UC and CD and to summarize recent evidence regarding the effect of new treatment options on those targets. It was also intended to overview the relationship between endoscopic and histological endpoints and IBD prognosis and to explore the unmet needs in this field.

## Search strategy

A narrative review was conducted to collect the endoscopic and histologic scores used for grading IBD. To assess the effectiveness of recent medication in achieving endoscopic response and remission, we conducted a search on CENTRAL and PubMed for all randomized-controlled trials (RCTs) published since 2020, focusing on the impact of pharmacological interventions on endoscopic response and remission in patients with UC and CD; the following terms were applied: ((endoscopic score) or (endoscopic response) or (endoscopic remission)) and ((UC) or (CD)) and (RCT).

## Endoscopic outcomes

### Ulcerative colitis

#### Are endoscopic response and remission achievable in UC?

Several studies have demonstrated that current ulcerative colitis (UC) medical therapies have the capability to induce favourable endoscopic outcomes.^12,13^ The summary of the RCTs published since 2020 is presented in Table 1.^14–27^ Endoscopic remission after induction was achieved between 2.6 percent (after 12 weeks of mirikizumab^18^) and 22.0 percent (after 12 weeks of olamkicept^25^) of the patients. On the other hand, from 14.6 percent (after 58 weeks of filgotinib^16^) to 30.6 percent (etrolizumab for 62 weeks^23^) were in endoscopic remission during maintenance. Endoscopic response was achieved in 13.1 percent (for the oral α_4_β_7_ antagonist PTG-100^20^) to 60.7 percent (for apremilast^27^) of patients after induction treatment. Regarding maintenance, improvement of endoscopic lesions has been reported to occur between 27.1 percent (with etrolizumab^26^) and 55.3 percent (with upadacitinib^14^) of the patients. All studied drugs demonstrated a positive effect on these outcomes when compared with the placebo, even though the differences were modest in some studies. The Mayo Endoscopic subscore (MES) was the most applied index, usually as a secondary outcome. MES is an easy-to-use tool, which assesses inflammation based on a 4-point scale (0–3)^28,29^; MH is defined as an MES of 0 or 1, depending on the studies. However, MES presents some limitations: it lacks formal validation, has a low sensitivity to change over time, and does not provide an accurate distinction between moderate and mild friability, which leaves room for subjectivity and interobserver variability.^28^

**Table 1.** Endoscopic response and remission achieved in randomized-controlled trials of Crohn’s disease and ulcerative colitis published since 2020.

Disease	Authors	Intervention and population	Intervention	Endoscopic score	Rate of endoscopic remission	Rate of endoscopic response
Crohn’s disease	Sandborn et al. (2020)^37^	Moderate to severe CD, inadequate response, or intolerance to IS or anti-TNF	UPA (3 mg, 6 mg, 12 mg, or 24 mg twice daily) or placebo; 16 weeks (induction)	SES-CD	UPA 48/183 (26.2 percent) versus placebo 0/37 (0.0 percent)	UPA 96/183 (52.5 percent) versus placebo 3/37 (8.1 percent)
	D’Haens et al. (2022)^38^	Moderate to severe CD, inadequate response, or intolerance to IS or biologics	RIS (600 mg or 1,200 mg) or placebo; 12 weeks (induction)	SES-CD	NR	RIS 364/1057 (34.4 percent) versus placebo 42/362 (11.6 percent)
	Ferrante et al. (2022)^39^	Moderate to severe CD, inadequate response, or intolerance to IS or biologics	RIS (180 mg or 360 mg) or placebo; 52 weeks (maintenance)	SES-CD	RIS 102/298 (34.2 percent) versus placebo 21/164 (12.8 percent)	RIS 140/298 (47.0 percent) versus placebo 36/164 (22.0 percent)
	Danese et al. (2022)^40^	Moderate to severe CD, inadequate response to IS or biologics	UST 90 mg; 48 weeks (maintenance)	SES-CD	Treat-to-target 25/219 (11.4 percent) versus standard-of-care 32/221 (14.5 percent)	Treat-to-target 83/219 (37.9 percent) versus standard-of-care 66/221 (29.9 percent)
	Sandborn et al. (2022)^41^	Moderate to severe CD, inadequate response, or intolerance to IS or biologics	GUS (200, 600, or 1,200 mg at week 0, 4, and 8); or placebo; 12 weeks (induction)	SES-CD	NR	GUS 66/185 (35.7 percent) versus placebo 7/61 (11.5 percent)
	Sands et al. (2022)^42^	Moderate to severe CD, inadequate response, or intolerance to IS or biologics	MIR (200, 600, or 1,000 mg every 4 weeks; or placebo; 12 weeks (induction)	SES-CD	MIR 20/127 (15.7 percent) versus placebo 1/64 (1.6 percent)	MIR 48/127 (37.8 percent) versus placebo 7/64 (10.9 percent)
	D’Haens et al. (2022)^43^	Moderate to severe CD, inadequate response, or intolerance to IS (25 percent had prior IFX exposure)	ADA—induction (high-dose [HD] 160 mg at week 0, 1, 2 and 3, 40 mg at week 4] or standard-dose [SD—160 mg at weeks 0, 80 mg at weeks 2, 40 mg at week 4] for 12 weeks; 40 mg eow for 56 weeks (maintenance)	SES-CD	HD 62/308 (20.1 percent) versus SD 54/206 (26.2 percent)	HD 132/308 (42.9 percent) versus SD 81/206 (39.3 percent)
	Sands et al. (2020)^44^	Moderate to severe CD, inadequate response, or intolerance to IS or biologics	GED-0301 (Mongersen): 160 mg orally; or placebo; 12 weeks (induction)	SES-CD	NR	GED-0301 10.1 percent (29/160) versus placebo 18.1 percent (49/483)
	Sandborn et al. (2023)^45^	Moderate to severe CD, inadequate response, or intolerance to IS or biologics	ETR 105 mg (at weeks 0, 4, 8, and 12) or 210 mg (weeks 0, 2, 4, 8, and 12) or placebo; 14 weeks (induction); or ETR 105 mg every 4 weeks; 52 weeks or placebo (maintenance)	SES-CD	NR	Induction: ETR 40/148 (27.0 percent) versus placebo 21/95 (22.1 percent) Maintenance: ETR 51/212 [24.1 percent] versus placebo 26/216 [12.0 percent]
Ulcerative colitis	Danese et al. (2022)^14^	Moderate to severe UC, inadequate response, or intolerance to aminosalicylates, corticosteroid, IS, or biologics	UPA 45 mg once daily or placebo for 8 weeks (induction). UPA (15 or 30 mg), or placebo for 52 weeks (maintenance)	MES	Induction: UPA 106/660 (16.0 percent) versus placebo 5/328 (1.5 percent) Maintenance: UPA 76/302 (25.1 percent) versus placebo 8/149 (5.3 percent)	Induction: UPA 266/660 (40.3 percent) versus placebo 25/328 (7.6 percent). Maintenance: UPA 167/302 (55.3 percent) versus placebo 22/149 (14.7 percent)
	Sandborn et al. (2021)^15^	Moderate to severe UC	OZA 1mg once daily for 10 weeks (induction) and for 52 weeks (maintenance)	MES	NR	Induction: OZA 117/429 (27.2 percent) versus placebo 25/216 (11.5 percent). Maintenance: OZA 105/230 (45.6 percent) versus placebo 60/227 (26.4 percent)
	Faegan et al. (2021)^16^	Moderate to severe UC, inadequate response to IS and bio-naïve (study A) or bio-exposed (B)	FIL (200 mg or 100 mg), or placebo once daily for 11 weeks (induction), or 58 weeks (maintenance)	MES	Induction: FIL 61/1069 (5.7 percent) versus placebo 8/279 (2.8 percent). Maintenance: FIL 54/371 (14.6 percent) versus placebo 13/187 (7.0 percent)	NR
	Sandborn et al. (2020)^17^	Moderate to severe UC, inadequate response to IS or anti-TNF	VEDO (108 mg SC every 2 weeks, or 300 mg IV every 8 weeks), or placebo for 52 weeks (maintenance)	MES	VEDO 46/160 (28.8 percent) versus placebo 7/56 (12.5 percent)	VEDO 89/160 (55.6 percent) versus placebo 12/56 (21.4 percent)
	Sandborn et al. (2020)^18^	Moderate to severe UC	MIR (50, 200 or 600 mg or placebo SC every 4 weeks for 12 weeks (induction). MIR 200 mg every 4 or 12 weeks or placebo (maintenance)	MES	Induction: MIR 5/186 (2.6 percent) versus placebo 1/63 (1.5 percent). Maintenance: MIR 20/93 (21.5 percent) versus placebo 1/13 (7.6 percent)	Induction: MIR 42/186 (22.5 percent) versus placebo 4/63 (6.3 percent). Maintenance: MIR 49/93 (52.6 percent) versus placebo 2/13 (15.3 percent)
	Sandborn et al. (2020)^19^	Moderate to severe UC	ETRA (1 or 2 mg) or placebo once daily for 12 weeks (induction)	MES	NR	ETR 33/102 (32.3 percent) versus placebo 10/54 (18.5 percent)
	Sandborn et al. (2021)^20^	Moderate to severe UC, inadequate response to IS and anti-TNF	PTG-100 (150, 300, 900 mg), or placebo once daily for 12 weeks (induction)	MES	NR	PTG-100 8/61 (13.1 percent) versus placebo 1/21 (4.8 percent)
	Rubin et al. (2021)^21^	Moderate to severe UC, patients naïve to anti-TNF	ETR (105 mg every 4 weeks), ADA (160 mg on day 1, 80 mg at week 2, and 40 mg at week 4, 6, and 8); or placebo for 10 weeks (induction)	MES	ETR 58/287 (20.2 percent) versus ADA 67/285 (23.5 percent) versus placebo 11/144 (7.6 percent)	ETR 115/287 (40.1 percent) versus ADA 108/285 (37.9 percent) versus placebo 38/144 26.3( percent)
	Peyrin-Biroulet et al. (2022)^22^	Moderate to severe UC, previously treated with anti-TNF	ETR (105 mg or placebo every 4 weeks for 14 weeks (induction) or 66 weeks (maintenance)	MES	Induction: ETR 66/384 (17.2 percent) versus placebo 9/95 (9.5 percent). Maintenance: ETR 26/112 (23.2 percent) versus placebo 13/114 (11.4 percent)	Induction: ETR 128/384 (33.3 percent) versus placebo 24/9 (25.3 percent). Maintenance: ETR 40/112 (35.7 percent) versus placebo 24/114 (21.1 percent)
	Vermeire et al. (2022)^23^	Moderate to severe UC, patients naïve to anti-TNF	ETR (105 mg every 4 weeks) or placebo for 62 weeks (maintenance)	MES	ETR 33/108 (30.6 percent) versus placebo 17/102 (16.7 percent)	ETR 41/108 (38.0 percent) versus placebo 23/102 (22.5 percent)
	Sandborn et al. (2023)^24^	Moderate to severe UC, inadequate response to IS or biologics	ETR (2 mg) or placebo once daily for 12 weeks (induction) or 52 weeks (maintenance)	MES	Induction: ETR 78/497 (15.7 percent) versus placebo 15/238 (6.3 percent). Maintenance: ETR 72/274 (26.2 percent) versus placebo 8/135 (5.9 percent)	Induction: ETR 164/495 (33.1 percent) versus placebo42/251(16.7 percent). Maintenance: ETR 99/262 (37.7 percent) versus placebo 16/135 (11.8 percent)
	Zhang et al., 2023^25^	Moderate to severe UC	OLA (300 or 600 mg) or placebo for 12 weeks (induction)	MES	OLA 13/59 (22.0 percent) versus placebo 1/29 (3.4 percent)	NR
	Danese et al., 2022^26^	Moderate to severe UC, patients naïve to anti-TNF	ETR (105 mg every 4 weeks) or IFX (5 mg/kg at 0, 2, and 6 weeks and every 8 weeks) thereafter for 10 (induction) and 52 weeks (maintenance)	MES	Maintenance: ETR 35/199 (17.6 percent) versus IFX 45/198 (22.7 percent) [no data for induction phase]	Induction: ETR 73/199 (36.7 percent) versus IFX 98/198 (49.5 percent). Maintenance: ETR 54/199 (27.1 percent) versus IFX 64/198 (32.3 percent)
	Danese et al., 2020^27^	Moderate to severe UC, patients naïve to biologics	APR (30 or 40 mg twice daily), or placebo for 12 weeks (induction)	MES	NR	APR 68/112 (60.7 percent) versus placebo 24/58 (41.4 percent)

Abbreviations: ADA, adalimumab; APR, apremilast; CD, Crohn’s disease; ETR, etrolizumab; ETRA, etrasimod; EOW, every other week; FIL, filgotinib; GUS, guselkumab; HD, high dose; IS, immunosuppressants; IFX, infliximab; IV, intravenous; MES, Mayo endoscopic score; MIR, mirikizumab; NR, not reported; OLA, olamkicept; OZA, ozanimod; RIS, risankizumab; SC, subcutaneous; SD, standard dose; SES-CD, simple endoscopic score for Crohn’s disease; TNF, tumour necrosis factor-alpha; UC, ulcerative colitis: UPA, upadacitinib; UST, ustekinumab; VEDO, vedolizumab; W, week(s).

#### Do endoscopic outcomes impact UC prognosis?

In a recent meta-analysis of seventeen studies (five of whom prospective) that included over 2,500 UC patients, MES 0 patients had a 52 percent lower risk of clinical relapse compared with those with MES 1 (29 percent versus 14 percent).^30^ The predictive ability of endoscopic outcomes has been also demonstrated for the ulcerative colitis endoscopic index of severity (UCEIS), a score that has already been prospectively validated.^31,32^ Indeed, UCEIS has demonstrated a capability of predicting the need for colectomy^33^ patients with acute severe UC with a UCEIS of 3 had a colectomy rate of 0 percent, while those with a UCEIS of at least 7 had a rate of 80 percent, after a median follow-up of eighteen months. UCEIS has also been reported to predict the response to biological treatment.^34^ More recently, a new index, the Paddington international virtual chromoendoscopy score in ulcerative colitis (PICaSSO), was prospectively validated.^35^ This index was able to accurately predict histological healing and long-term remission. Indeed, patients with a PICaSSO score ≤ 3 were less prone to UC clinical relapse than those with PICaSSO > 3 (hazard ratio ~0.20 at 6 and 12 months after colonoscopy).^36^

### Crohn’s disease

#### Are endoscopic response and remission achievable in Crohn’s disease?

The regression or disappearance of lesions in ileocolonoscopy of Crohn’s disease (CD) patients has been regarded as endoscopic remission or MH and is, now, considered a therapeutic target. Over time, several scoring systems have been developed to increase the objectivity of endoscopic evaluation. The simple endoscopic score for CD (SES-CD) was introduced in 2004. A relevant drawback of SES-CD is the lack of clarity regarding its validation, responsiveness, and reliability in predicting outcomes in CD. Indeed, its thresholds are mostly arbitrary, corresponding endoscopic response, usually, to a reduction of at least 50 percent in baseline SES-CD, and remission to a score of up to 2 or 3 points. Since 2020, nine RCTs^37–45^ on the effect of CD therapies on endoscopic outcomes was published; all the studies have used the SES-CD index (Table 1). Even though the interventions were diverse (including drugs targeting interleukins,^38–41^ Janus-kinase inhibitors,^37^ anti-tumor necrosis factor,^43^ or integrins^45^), it seems clear that currently approved drugs and those on the pipeline allow reaching endoscopic outcomes in a significant proportion of patients with moderate to severe CD; the rates of endoscopic response and remission were between 30 percent and 50 percent and 15 percent and 20 percent, respectively, after induction therapy. The achievement of endoscopic response and remission during maintenance was recently evaluated in four studies.^39,40,43,45^ The percentages of response were between 24.1 percent (after 52 weeks of etrolizumab^45^) and 47.0 percent (52 weeks of risankizumab^39^), while those if endoscopic remission ranged between 14.5 percent (for ustecinumab^40^) and 34.2 percent (for risankizumab^39^). Over the last decade, differences between ileal and colonic Crohn’s disease in terms of therapeutic responses have been highlighted. A recent post-hoc analysis of an RCT of patients who received induction and maintenance therapy with anti-TNF agents found that endoscopic healing (evaluated using modified SES-CD) of the small bowel occurred in 36 percent, while 79 percent of the patients demonstrated endoscopic healing in the colon.^46^ Interestingly, all patients with small bowel healing had concurrent colonic endoscopic healing. This information was in line with the described by the post-hoc analyses of the SONIC and TAILORIX trials, where it was also highlighted that the degree of endoscopic inflammation at baseline did not affect the likelihood of achieving endoscopic remission.^47,48^ Of the RCTs published since 2020, only one (comparing standard- versus high-dose adalimumab^43^) provided data on the achievement of endoscopic outcomes depending on the location. In this study, the attainment of endoscopic response at week 12 was superior for patients with colonic activity only (40.3 percent vs. 32.7 percent).^43^ Also, the effect of the high- versus standard-dose therapy was larger for patients with ileal versus colonic disease (delta of 19.0 vs. 6.1).^43^

#### Do endoscopic outcomes impact CD prognosis?

A meta-analysis of 12 studies, in prospective cohorts with active CD, has demonstrated that patients who achieved MH, at first assessment (at least 1 month after study onset, but 6 months prior to the last follow-up), were more likely to achieve long-term clinical remission (at week 50), had lower rates of CD-related surgery, and were more prone to maintain long-term MH.^49^ In this meta-analysis, the SES-CD score was used in eight studies and the cutoffs applied to grade endoscopic changes varied (MH defined as SES-CD = 0 only in half). In 2017, another meta-analysis^50^ of five studies revealed that small-bowel MH, assessed through capsule endoscopy and graded using the Niv or the Lewis score, was able to predict long-term clinical remission (up to 24 months). In addition, in a post-hoc analysis of the SONIC clinical trial, participants with an endoscopic response (decrease in SES-CD or CD endoscopic index of severity [CDEIS] of at least 50 percent, at week 26) were significantly more likely to achieve a 1-year corticosteroid-free clinical remission.^51^ In line with this, a follow-up analysis for the CALM study showed a correlation between the achievement of endoscopic remission (CDEIS of <4 with no deep ulcers), in individuals with early CD and a reduced risk of disease progression over a median duration of 3 years.^52^ However, in this study, deep remission (endoscopic and clinical remission) predicted prognosis with a higher effect size. More recently, the prognostic value of endoscopy was ascertained using another score, the modified multiplier simple endoscopic score for CD (MM-SES-CD). Compared with SES-CD, this scoring system has the advantage of detecting disease heterogeneity,^53^ by attributing varying weights to each individual parameter of SES-CD, according to their prognostic values for achieving endoscopic remission.^53^ A reduction ≥40 percent in MM-SES-CD, after biologic initiation, was found to be significantly more specific at predicting endoscopic remission after one year compared with SES-CD (95 percent vs. 63 percent, respectively).^53^

## Histological outcomes

### Ulcerative colitis

#### Are histological response and remission achievable in UC?

While the value of histology in UC has been recognized since early clinical trials,^54,55^ attention has been directed toward formal histological scoring only in recent years. Even though the concept of MH was initially defined by endoscopic evaluation alone, since 2016, guidance from the Food and Drug Administration recommends that it should encompass also histological healing.^56^ However, the histological scoring of UC is a complex process comprising twenty six distinct histopathological scoring systems.^57^ In this setting, the European Crohn’s and Colitis Organization (ECCO) has recommended the application of the Robarts histopathology index (RHI) and the Nancy histopathology index (NHI) in clinical trials,^10^ both already validated; NHI has been also recommended for observational studies and clinical practice. RHI includes four items related to chronic inflammation, neutrophilic inflammation, and surface epithelial injury, ranging from 0 to 33^58^; histological remission is defined as RHI ≤ 3 and histological response as RHI ≤ 9. On the other hand, NHI assesses lamina propria chronic inflammation, neutrophilic inflammation, and surface damage (erosion/ulcers) on a scale that ranges from 0 to 4.^59^ Histological remission is defined as an NHI of 0 and histological response as an NHI ≤ 1. This index has a strong correlation with RHI, good interobserver variability, and adequate response to change after the treatment.^3,60^ Another system that is deemed suitable to evaluate the histologic activity of UC is the Geboes score (GS). GS ranges from 0 to 5.4; being histological remission defined as GS ≤ 2.0 and histological response as GS ≤ 3.0.^3^ This score has not been fully validated^8,57^ and its complexity limits its use in daily clinical practice. It is also important to acknowledge that it was not explicitly intended for quantifying changes in histologic activity because of its hierarchical scoring system.^57^

Real-world evidence has shown that drugs currently indicated for UC, including infliximab,^12^ adalimumab,^13^ vedolizumab,^61^ and ustekinumab,^62^ are able to induce histological remission in up to 45 percent of the patients. To overview the ability of most recent therapies to achieve histological outcomes, we have conducted a search for RCTs, published since 2020, that examined the impact of drugs on histological endpoints in UC; the search included the terms “((histologic score) or (histologic response) or (histologic remission)) and (ulcerative colitis) and (RCT)”. Overall, ten studies were identified^14–16,19–23,27,63^ (Table 2). As reported in a previous meta-analysis, the most used score was the GS.^8^ On the other hand, NHI was applied in three studies,^21–23^ while two studies resorted to RHI.^20,63^ In general, all studied drugs allowed achieving histological outcomes more frequently than placebo, yet with variable magnitudes of effect. The percentages of histologic response ranged from 24.4 percent to 58.6 percent on induction (for adalimumab^63^ and upadacitinib,^14^ respectively) and from 25.6 percent to 45.7 percent on maintenance (for adalimumab^63^ and vedolizumab^63^).

**Table 2.** Histological response and remission in randomized-controlled trials published since 2020.

Disease	Authors	Intervention and population	Intervention	Histological score	Rate of histological remission	Rate of histological response
CD	Magro et al. (2022)^61^	Moderate to severe CD	MIR (200, 600, or 1000 mg) every 4 weeks, or placebo; 12 weeks (induction); MIR every 4 weeks; 52 weeks (maintenance)	GHAS	Induction: MIR 30/158 (19.0 percent), maintenance: MIR 37/158 (23.4 percent) [data on placebo NR]	Induction: MIR 74/158 (46.8 percent), maintenance: MIR 74/158 (46.8 percent) [data on placebo NR]
Ulcerative colitis	Danese et al. (2022)^14^	Moderate to severe UC, inadequate response to IS, or biologics	UPA 45 mg once daily or placebo for 8 weeks (induction); UPA (15 or 30 mg) or placebo for 52 weeks (maintenance)	GS	–	Induction: UPA 387/660 (58.6 percent) versus placebo 78/328 (23.7 percent)
	Sandborn et al. (2021)^15^	Moderate to severe UC	OZA 1mg once daily for 10 weeks (induction) and for 52 weeks (maintenance)	GS	Induction: OZA 78/429 (18.1 percent) versus placebo 16/216 (7.4 percent). Maintenance: OZA 77/230 (33.4 percent) versus placebo 37/227 (16.2 percent)	NR
	Feagan et al. (2021)^16^	Moderate to severe UC, inadequate response to IS and biologic naïve (study A) or inadequate response to anti-TNF and VEDO (study B).	FIL (100 or 200 mg), or placebo once daily for 11 weeks (induction) and for 58 weeks (maintenance)	GS	Induction: FIL 243/1069 (22.7 percent) versus placebo 34/279 (12.1 percent); maintenance: FIL 124/371 (33.4 percent) versus placebo 29/187 (15.5 percent)	NR
	Sandborn et al. (2020)^19^	Moderate to severe UC	ETRA (1 or 2 mg), or placebo once daily for 12 weeks (induction)	GS	ETR 13/90 (14.4 percent) versus placebo 3/49 (6.1 percent)	ETR 23/90 (25.5 percent) versus placebo 5/49 (10.2 percent)
	Peyrin-Biroulet et al. (2021)^60^	Moderate to severe UC, inadequate response to anti-TNF or anti-TNF naïve	VEDO (300 mg every 8 weeks) or ADA 40 mg every 2 weeks for 50 weeks (induction and maintenance)	GS and RHI	Induction: GS—VEDO 64/383 (16.7 percent) versus ADA 28/386 (7.2 percent); RHI—VEDO 98/383 (25.5 percent) versus ADA 62/386 (16.0 percent). Maintenance: GS—VEDO 112/383 (29.2 percent) versus ADA 32/386 (8.3 percent); RHI—VEDO 144/383 (37.5 percent) versus ADA 77/386 (19.9 percent)	Induction: GS—VEDO 173/383 (45.2 percent) versus ADA 118/386 (30.6 percent); RHI—VEDO 142/383 (37.3 percent) versus ADA 94/386 (24.4 percent). Maintenance: GS—VEDO 175/383 (45.7 percent) versus ADA 119/386 (30.8 percent); RHI—VEDO 162/383 (42.3 percent) versus ADA 99/386 (25.6 percent)
	Sandborn et al. (2021)^20^	Moderate to severe UC, inadequate response to IS and anti-TNF	PTG-100 150 mg, 300 mg, 900 mg, or placebo once daily for 12 weeks (induction)	RHI	PTG-100 8/33 (24.2 percent) versus placebo 0/13 (0.0 percent)	NR
	Rubin et al. (2021)^21^	Moderate to severe UC, patients naïve to anti-TNF	ETR (105 mg every 4 weeks), ADA (160 mg on day 1, 80 mg at week 2, and 40 mg at weeks 4, 6, and 8); or placebo for 10 weeks (induction)	NHI	ETR 84/228 (36.8 percent) versus ADA 84/230 (36.5 percent) versus placebo 23/124 (18.5 percent)	NR
	Peyrin-Biroulet et al. (2022)^22^	Moderate to severe UC, previously treated with anti-TNF	ETR SC 105 mg or placebo every 4 weeks for 14 weeks (induction) and for 66 weeks (maintenance)	NHI	Induction: ETR 92/310 (29.7 percent) versus placebo 20/80 (25.0 percent). Maintenance: ETR 28/91 (30.8 percent) versus placebo 13/92 (14.1 percent)	NR
	Vermeire et al. (2022)^23^	Moderate to severe UC, patients naïve to anti-TNF	ETR SC 105 mg every 4 weeks or placebo for 62 weeks (maintenance)	NHI	ETR 36/85 (42.4 percent) versus placebo 17/78 (21.8 percent)	NR
	Danese et al. (2020)^27^	Moderate to severe UC, patients naïve to biologics	APR(30 or 40 mg) twice daily, or placebo for 12 weeks (induction)	GS	APR 48/112 (42.8 percent) versus placebo 17/58 (29.3 percent)	NR

Abbreviations: ADA, adalimumab; APR, apremilast; CD, Crohn’s disease; ETR, etrolizumab; ETRA, etrasimod; FIL, filgotinib; GS, Geboes score; MIR, mirikizumab; NHI, Nancy histopathology index; NR, not reported; OZA, ozanimod; RHI, Robarts histopathology index; TNF, tumour necrosis factor-alpha; UC, ulcerative colitis; UPA, upadacitinib; VEDO, vedolizumab; W, week(s).

#### Do histological outcomes impact UC prognosis?

The presence of neutrophilic inflammation within the lamina propria and epithelium defines histological activity,^3^ while its absence corresponds to histological remission.^7^ In a meta-analysis of twenty studies (twelve prospective), patients in endoscopic healing or remission (MES 0) and histological remission (evaluated using different scores) had a 61 percent lower risk of clinical relapse compared with those with persistent histological activity (14 percent vs. 5 percent).^30^ Another meta-analysis of seventeen studies has shown that patients in endoscopic remission (MES of 0 or 1) but with persistent histological activity had a threefold increased risk of presenting UC relapse.^64^ In these meta-analyses, a more stringent target was associated with a better prognosis, as also shown by Magro et al.^65^ Indeed, these authors showed that GS is an independent risk factor in the 36 months after biopsy, with disease progression occurring earlier and more frequently in patients with higher GS. In detail, 26 percent of patients with GS ≤2B had progressive disease compared with 50 percent of those with GS > 4.0. More recently, it has been shown that the PICaSSO histologic remission index (PHRI), a new score based on the presence or absence of neutrophils (and recently externally validated), presented the strongest correlation to endoscopic activity and excellent prediction of clinical outcomes, among several histological grading systems. In fact, patients with PHRI above 0 had significantly more events associated with UC exacerbations than those with PHRI = 0 (49 percent vs. 14 percent, at month 12).^66^ The prognostic role of histologic remission was again demonstrated by Rath et al.^57^. Indeed, patients with RHI up to three points or NHI up to 1 had a significantly higher likelihood of not presenting UC-related adverse events (disease flares, IBD-related hospitalization or surgery, initiation or dose escalation of systemic steroids, immunosuppressants, or targeted therapy) during follow-up. Despite this evidence, it remains to be understood whether less rigorous monitoring may be proposed for UC patients in histologic remission.^57^

### Crohn’s disease

#### Are histological response and remission achievable in CD?

The presence of neutrophilic epithelial infiltrate is, as in UC, a straightforward manner to, dichotomously, evaluate histologic activity in CD. Still, several other changes (like basal plasmacytosis, chronic inflammatory infiltrate in the lamina propria, and presence of erosions and ulcers)^3^ have been reported to have prognostic relevance. Over the decades, several scoring systems have been developed, with variable ranges and remission/activity cutoffs defined only for some (Geboes,^67^ Naini and Cortina,^68^ Global Histologic Disease Activity Score [GHAS],^69^ Regueiro,^70^ and Saverymuttu^71^ scores); a few systems were developed for specific sites (ileal^72^ [Drews Score], colonic^73^ [Gomes Score], or rectal^74^ [Ward and Webb] samples). One notable advantage of GHAS lies in the distinct evaluation of neutrophil infiltration in the lamina propria and of a persistent chronic inflammatory response, which remains after treatment. While extensive prospective studies have not yet validated this scoring system, it remains the most employed measure in clinical trials focused on assessing microscopic activity in CD. Likewise, none of the other scores has been validated nor is commonly used in routine practice.

As for UC, we searched for RCTs evaluating histological response and remission, published since 2020. However, the search on two databases (CENTRAL and PubMed) retrieved no results. A search among the abstracts presented at ECCO meetings since 2020 identified a trial (SERENTIY; NCT02891226) that evaluated the efficacy of mirikizumab^75^ on histologic outcomes, ascertained through GHAS. Almost half of the patients showed histological response, after 12 and 52 weeks of therapy, while around 20 percent achieved remission. These results suggest that histological outcomes may be a realistic goal also in CD, as highlighted in a metanalysis of older studies.^76^ Further RCTs evaluating the impact of therapeutics on CD histopathology are awaited.

#### Do histological outcomes impact CD prognosis?

Data on the evaluation of the feasibility and impact of achieving histological outcomes in CD patients is limited and restricted to retrospective studies. This is mostly related to the patchy and transmural nature of CD.

In a study by Christensen et al. with hundred patients with ileal CD, the presence of histological remission (defined as GHAS = 0), but not endoscopic remission (no erosions nor ulceration), was associated with a reduction in clinical relapse, medication escalation, and need for corticosteroids.^77^ Conversely, Hu et al.^78^ did not observe a notable difference in the risk of relapse or quality of life in CD patients in endoscopic remission (SES-CD of 0), regardless of whether they achieved histological remission (defined as an absence of active or quiescent disease) or not. However, in this cohort, the duration of endoscopic remission, prior disease activity, and therapeutics were not assessed. In 2021, a study comprising 215 CD patients with histological assessment showed that, over a 2-year follow-up period, individuals who were in clinical, endoscopic, and histological remission (no evidence of disease or quiescent disease, per the Nancy index) had a 43 percent reduced risk of treatment failure compared with those who had ongoing histologic activity.^79^ However, no significant differences were observed in the magnitude of the benefit of histological remission based on the depth of remission (complete normalization vs. quiescent disease).

## Unmet needs for pursuing endoscopic outcomes

Even though the evaluation of endoscopic endpoints is of utmost relevance for drug development, their systematic evaluation and reporting in everyday clinical practice are still suboptimal, particularly for CD. As such, the systematic use of endoscopic scores, such as SES-CD, outside clinical research settings, should be promoted, to improve the estimation of disease activity and prognosis. This will impact clinical decisions, including treatment escalation and de-escalation.

In this setting, research efforts are being directed at developing tools to increase clinician adherence to endoscopic scores. For instance, the novel simplified endoscopic mucosal assessment for CD (SEMA-CD) score was recently derived from the SES-CD with this objective.^80^ Even though simplified approaches have their appeal, some knowledge gaps may be filled by understanding patterns of endoscopic healing and focusing on challenging and harder-to-heal bowel segments. In addition, updated endoscopic scoring systems to predict postoperative recurrence following ileocecal resection are warranted. Indeed, the Rutgeerts score was developed during the end-to-end anastomosis era and, as such, may not be appropriate for modern surgical techniques. For example, patients who undergo side-to-side anastomosis may have a higher number of ileal lesions but fewer anastomotic lesions while an ileal body may be present near the inlet of the neoterminal ileum.

Regarding UC, even though endoscopic activity is often evaluated using MES, in real-world practice and in clinical trials, the use of more robust and validated indexes (such as the UCEIS^31^ and the ulcerative colitis colonoscopic index of severity^81^) remains suboptimal.

Several artificial intelligence (AI) tools are being developed to score IBD endoscopic lesions, particularly in the setting of UC. Indeed, several deep learning algorithms have been described to be able to predict UC severity (graded using the MES, UCEIS or, more recently, PICaSSO^82,83^) from full-length colonoscopy videos. Concerning CD, most studies focused on capsule endoscopy readings for automated endoscopic grading.^84^ It is anticipated that the application of AI tolls will increase objectivity, reducing interindividual subjectivity and increasing adherence to scores.

## Unmet needs for using histology as a target

Regarding CD, it is mandatory to validate the existing histopathological scores (particularly GHAS) and promote their incorporation as secondary endpoints in clinical trials. Indeed, since 2020, only one RCT did so.^75^ Furthermore, investigating the prognostic significance of histological remission through prospective studies is also warranted. Such prognostic information would be particularly valuable within the context of the treatment cycling concept,^85^ in which the discontinuation of biological treatment may be considered for specific patients. In addition, it is not clear if histology is the key tool to monitor relapsing episodes, following treatment discontinuation. Also, even though data on the correlation between imaging scores (particularly regarding intestinal ultrasound) and endoscopic ones are starting to emerge,^86^ the correlation with histological scores remains uncovered.

Regarding UC, although evidence supports that histological remission has prognostic relevance, several aspects hamper its utilization as a formal target. First, doubts persist regarding the significant added value of histology in patients who present clinical and/or endoscopic activity and/or present high values of fecal calprotectin. Second, the costs of this evaluation are high. Third, a standardized and uniform definition for histological remission is urgent but is still lacking. The use of simpler scores (based solely on the presence of neutrophils, as the PHRI), the implementation of formal training, and centralized reading may improve adherence, reporting quality, and patient monitoring.^57^ The impact of histological remission on monitoring frequency remains also underexplained.

Finally, a quick note about the application of AI algorithms for the histopathological evaluation in IBD. Recently, a convolutional neural network-based model was developed to classify the histologic features of patients with UC.^66^ This algorithm categorizes patients as being on histological remission or non-remission, depending on the presence of neutrophils in whole slide images.^66^ The overall performance of this model was good, with values of sensitivity and specificity of 81 percent and 93 percent in the validation set.^66^ Other research groups have also developed similar models, achieving comparable diagnostic performances.^87^ Conversely, the application of automation for CD histological assessment is less studied. The single study found used a deep-learning model for the analysis of surgical specimens and to predict CD postoperative recurrence; its performance was outstanding (area under the curve of 0.995).^88^ These technical innovations will eventually lead to a standardized assessment of disease activity, which, up to now, is highly subjective and burdened by interobserver variability.

To finalize, future studies may provide further clarification on the feasibility and usefulness of molecular healing as a treatment target for IBD. Indeed, a recently published prospective study^2^ involving CD and UC patients in clinical remission found that barrier healing, evaluated through confocal laser endomicroscopy, was more predictive of significant adverse outcomes than histologic remission.

## Data Availability

The data underlying this article are available in the article and in its online supplementary material.
